# Biomarkers as Predictors of Mortality in Sepsis and Septic Shock for Patients Admitted to Emergency Department: Who Is the Winner? A Prospective Study

**DOI:** 10.3390/jcm13195678

**Published:** 2024-09-24

**Authors:** Sonia Luka, Adela Golea, Raluca Mihaela Tat, Eugenia Maria Lupan Mureșan, George Teo Voicescu, Ștefan Cristian Vesa, Daniela Ionescu

**Affiliations:** 1 Department 6 Surgery, Discipline of Emergency Medicine, Iuliu Hatieganu, Faculty of Medicine, University of Medicine and Pharmacy, 3-5 Clinicilor Street, 400347 Cluj-Napoca, Romania; raluca.tat@umfcluj.ro (R.M.T.); eugenia.lupan@elearn.umfcluj.ro (E.M.L.M.); 2 Clinical Emergency County Hospital, 3-5 Clinicilor Street, 400347 Cluj-Napoca, Romania; george.voicescu@uniupo.it; 3 CRIMEDIM—Center for Research and Training in Disaster Medicine, Humanitarian Aid and Global Health, Università del Piemonte Orientale, 28100 Novara, Italy; 4 Department 1 Functional Sciences, Discipline of Pharmacology, Toxicology and Clinical Pharmacology, Faculty of Medicine, Iuliu Hatieganu University of Medicine and Pharmacy, 23 Marinescu Street, 400337 Cluj-Napoca, Romania; stefan.vesa@umfcluj.ro; 5 Department 6 Surgery, Discipline of Anaesthesia and Intensive Care I, Faculty of Medicine, Iuliu Hatieganu University of Medicine and Pharmacy, 19-21 Croitorilor Street, 400162 Cluj-Napoca, Romania; daniela_ionescu@umfcluj.ro; 6 Department of Anaesthesia and Intensive Care, The Regional Institute of Gastroenterology and Hepatology, Prof. Dr. Octavian Fodor, 19-21 Croitorilor Street, 400162 Cluj-Napoca, Romania; 7 Research Association in Anaesthesia and Intensive Care (ACATI), 400394 Cluj-Napoca, Romania; 8 Outcome Research Consortium, Cleveland, OH 44195, USA

**Keywords:** biomarkers, prognostic, mortality, sepsis, suPAR

## Abstract

**Background/Objectives**: Sepsis and septic shock remain significant contributors to high early mortality rates among patients admitted to the emergency department (ED). The objective of this study was to identify among newer biomarkers those with the highest sensitivity in early mortality prediction. **Methods**: This prospective, unicentric, observational study enrolled 47 adult patients admitted to the ED between November 2020 and December 2022. This study monitored the kinetics of the older and newer biomarkers, including azurocidin (AZU1), soluble triggering receptor expressed on myeloid cells (sTREM), soluble urokinase-type plasminogen activator receptor (suPAR), high-sensitivity C-reactive protein (hsCRP), procalcitonin (PCT), and interleukin-6 (IL-6), and their capacity in predicting mortality. **Results**: SuPAR showed the most significant predictive utility for early prognosis of mortality in the ED, with an area under the curve (AUC) of 0.813 (95% CI: 0.672 to 0.912), a cutoff value > 8168 ng/mL, sensitivity of 75%, and specificity of 81.48% (*p* < 0.001). IL-6 and PCT showed comparable prognostic accuracy, whereas hsCRP and AZU1 demonstrated lower predictive performance. **Conclusions**: In our study, suPAR, IL-6, and PCT showed good predictive value for short-term mortality in sepsis and septic shock patients.

## 1. Introduction

Despite the advances in modern medicine, sepsis and septic shock remain two critical conditions that represent significant challenges for EDs worldwide. These conditions often result in substantial patient morbidity and high mortality, particularly affecting vulnerable populations such as the elderly, immunosuppressed patients, and those with multiple comorbidities [1]. The management of sepsis and septic shock frequently requires a multidisciplinary approach, which involves the contributions of several medical specialties along with the establishment of immediate care and life support measures in the ED and, last but not least, often requires hospitalization in intensive care units (ICUs) for prolonged hospitalization periods [2]. Additionally, sepsis and septic shock represent a significant healthcare logistics challenge due to their expensive treatment costs, the need for further investigation, and long hospital stays [3].

Sepsis is a complex condition that is difficult to diagnose due to its diverse causative agents, variable disease progression, and the varied stages at which patients present to the emergency department (ED). These challenges may delay targeted treatments, adversely affecting patient outcomes [4]. While clinical signs are often non-specific, biomarkers offer potential for earlier sepsis detection and treatment initiation. However, accurately identifying the infection type and distinguishing infection-related inflammation remain difficult in many ED cases [5].

Multiple alert systems, scores, and interventions are now available for early detection of sepsis. However, there is currently no golden standard method that may reliably predict outcomes for all patients and guide therapy from early stages of diagnosis. Since EDs are the primary assessment points of these patients, early recognition and initiation of appropriate therapeutic measures based on the identification of highly sensitive and specific biomarkers, either alone or in combination with novel prognostic scores, could potentially improve long-term outcomes in the future [4].

High-sensitivity C-reactive protein (hsCRP), procalcitonin (PCT), soluble urokinase-type plasminogen activator receptor (suPAR), soluble triggering receptor expressed on myeloid cells (sTREM), interleukin-6 (IL-6), and azurocidin (AZU 1) are biomarkers commonly used to assess sepsis severity and predict patient outcomes.

While hsPCR [5,6,7] indicates systemic inflammation, PCT [8,9,10] aids in early diagnosis, risk stratification, and antibiotic management. Among the newer biomarkers, suPAR [11,12,13] correlates with immune activation, while sTREM [12,14,15] plays a significant role in innate immunity.

IL-6 [9,16,17] is widely used for assessing inflammatory activity, and AZU1 [18,19,20] is a novel biomarker used for sepsis prognosis.

Our study investigated these biomarkers, measured at three critical time points: at admission and first and second days post-admission, to assess their potential to predict short-term mortality with the highest accuracy. We chose this approach considering that patients are admitted to the ED at different stages of sepsis with varying degrees of severity, making the initial measurement of these biomarkers potentially unreliable.

## 2. Materials and Methods

### 2.1. Study Design and Setting

This observational, prospective, longitudinal and analytical study was carried out in Cluj-Napoca County Emergency Clinical Hospital’s ED, a tertiary hospital with academic activity and 1500 beds. This study included consecutive adult patients with sepsis or septic shock who were admitted to the ED between 1 November 2020, and 1 December 2022. Given that this study was conducted during the COVID-19 pandemic, recruitment of patients followed government regulations and local medical protocols regarding access to hospitalized patients.

This study adhered to the ethical principles and standards of clinical practice as stipulated in the Declaration of Helsinki [21] as well as EU legislation [22]. Patients above 18 years old were prospectively recruited and provided written informed consent themselves or via their legal representative within the first hour of arrival in the ED. This study was approved by the Ethics Committee of Iuliu Hațieganu University of Medicine and Pharmacy (Approval No. 139/30 March 2020) and the ECs of participating hospitals: Cluj Emergency County Hospital (Approval No. 5416/10 25 February 2020), Infectious Diseases Cluj-Napoca (Approval No. 6010/14 April 2021), and Clinical Institute of Urology and Renal Transplantation Cluj-Napoca (Approval No. 03/02 February 2021).

### 2.2. Study Design

Adult patients (over 18 years old and under 90 years) admitted to the ED for sepsis or septic shock, as defined by The Third International Consensus Definitions for Sepsis and Septic Shock—Sepsis 3, “Sepsis is defined as life-threatening organ dysfunction caused by a dysregulated host response to infection and an increase in the Sequential Organ Failure Assessment (SOFA) score of 2 points or more. Septic shock can be clinically identified by a vasopressor requirement to maintain a mean arterial pressure of 65 mm Hg or greater and serum lactate level greater than 2 mmol/L (>18 mg/dL) in the absence of hypovolemia” [23], were enrolled within one hour of presentation and after obtaining informed consent.

This is a planned sub-study focused on the dynamics of biomarkers in patients who survived for at least three days following presentation to the ED.

Exclusion criteria: patients who survived less than 48 h after admission, patients under 18 or over 90 years of age, patients with neoplastic disease, patients with other acute illnesses more severe than sepsis (other types of shock, cardiac arrest, the need of urgent surgery), pregnant women, incomplete data, or those who refused to participate in this study.

Upon arrival in the ED (T_0_) and after informed consent, each patient underwent a prospective evaluation, which included clinical assessment, vital signs monitoring, laboratory tests, and routine blood work. The same assessment was repeated at 24 h (T_24_) and 48 h (T_48_). A panel of six biomarkers (IL-6, hsPCR, PCT, AZU1, suPAR, and sTREM) was examined following admission at three time points (T_0_, T_24_, T_48_). Patients were monitored for 28 days following their referral to the ED, until either hospital discharge or death, if these events occurred within a 28-day period. Patients’ status was evaluated by a follow-up phone call at 28 days. Patients were categorized into two groups based on their outcomes at 28 days: survivors and non-survivors.

The primary objective was to evaluate the dynamics of this new set of biomarkers during the 48 h in sepsis or septic shock patients diagnosed in the ED. The secondary objective was to assess and compare their ability to predict 28-day mortality in these patients.

Medical management in the ED during this study was not based on the results of the novel inflammatory markers described in this manuscript but according to standard practice. Patients were admitted to the hospital and treated according to standard protocols, which included fluids, vasopressors, and wide spectrum antibiotics, in compliance with Surviving Sepsis Campaign recommendations [1].

Patients in our study were transferred from the ED to the ICU within an average of 6 h. Those with a mean arterial pressure (MAP) below 65 mmHg were treated in the ED with norepinephrine and fluid resuscitation. If hypotension persisted despite reaching a maximum noradrenaline dose of 0.5–0.7 µg/kg/min as recommended by protocols, vasopressin was administered in the ICU as an adjunct vasoconstrictor. For patients experiencing cardiac dysfunction due to sepsis or pre-existing heart disease, dobutamine was also associated.

### 2.3. Data Collection

The collected data included baseline demographic information, medical history, clinical evaluation, signs and symptoms, vital signs, laboratory testing (cultures and imaging), type of oxygenotherapy, medication and fluids administered during the ED stay and hospital stay, and survival status at 28 days. The laboratory tests included routine blood tests such as complete blood count (CBC), arterial blood gases (ABG), liver function, kidney function, coagulation, and blood cultures.

### 2.4. Sample Collection and Biomarker Assays

Blood samples for biomarker analysis were collected by ED nurses during the first hour of patient presentation, with informed consent. The samples were drawn using 5 mL serum separator tubes containing a clot activator and left to remain for 30 min at room temperature. Then they were centrifuged at 1000× *g* for 15 min. Plasma was transferred to a 1 mL Eppendorf tube and kept at −80 °C until assay. The samples were processed at the Cluj County Hospital laboratory. Hemolyzed samples were redrawn. The following Enzyme-Linked Immunosorbent Assay (ELISA) kits were used for biomarker analysis (BioVendor—Laboratorni medicina a.s., Karasek 1767/162100 Brno, Czech Republic). Biomarkers were evaluated using the Sandwich-ELISA immunoassay method. Heidolph Shaker Titramax 100 (Heidolph Instruments GmbH & Co. KG, Schwabach, Germany), ELISA Spectrophotometer (LabSystems Multiskan Plus LabSystems, Helsinki, Finland), and Autoanalyzer ELISA Personal Lab (ADALTIS, Rome, Italy) were among the analytical tools utilized. Measurements were made in accordance with the manufacturer’s instructions.

### 2.5. Statistical Analysis

Statistical analysis was performed using MedCalc^®^ Statistical Software version 22.021 (MedCalc Software Ltd., Ostend, Belgium; https://www.medcalc.org; accessed on 1 June 2024). The sample size was determined based on an initial small study group (n = 5 patients in each group). IL-6 mean values were 249.6 pg/mL in the survival group and 653.9 pg/mL in the non-survival group. A sample size of 16 patients per group was calculated in order to achieve a power of 80% and a level of significance of 5%. A 25% increase in sample size was added to compensate for eventual incomplete data and dropouts. We calculated sample size based on IL-6 levels considering that this is the most used biomarker in sepsis.

Quantitative data normality was assessed using the Shapiro–Wilk test. Quantitative data were expressed as median and (25th–75th) percentiles for non-normally distributed data or means ± standard deviation for normally distributed data. Qualitative data were characterized by frequency and percentage. For the biomarkers and score, we calculated the area under the curve (AUC) using the trapezoidal method with measurements from 0 to 24 h and 0 to 48 h. The differences between groups were verified with Mann–Whitney (for non-normally distributed data), Student *t*-test (normally distributed data), or chi-square test. Receiver operating characteristic (ROC) analysis was performed to determine a cutoff value for the association of several quantitative variables and mortality. The Youden index was used to determine the ideal cutoff threshold, ensuring an optimal balance of sensitivity and specificity. A *p*-value of less than 0.05 was considered statistically significant.

## 3. Results

**Figure 1:**
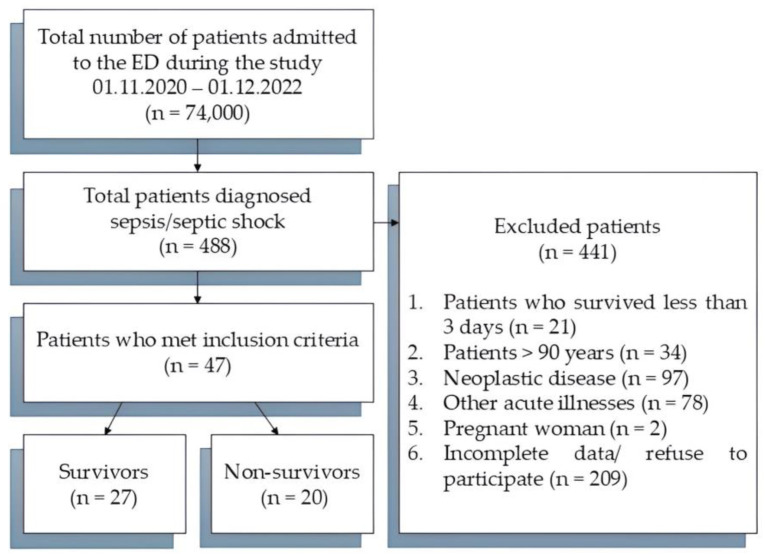
STROBE diagram of patient inclusion/exclusion criteria.

During the study period, 488 patients with sepsis or septic shock were referred to the ED. Of these, 47 patients met the eligibility criteria and were included in this study (Figure 1).

The overall 28-day survival rate was 57.44%. The median age of patients in our study was 71.5 years for survivors and 74.5 years for non-survivors.

85% of non-survivors and 55% of survivors had septic shock within the first 48 h of admission. Norepinephrine was the preferred vasopressor for all these patients.

Patients with altered mental status, as indicated by a lower GCS score, were associated with an increased mortality rate. As expected, the clinical prediction scores (SOFA, APACHE II, SAPS II) effectively discriminated between survivors and non-survivors.

Other predictors of 28-day mortality included tachycardia with a ventricular rate exceeding 110 bpm, lactate levels higher than 2 mmol/L, FiO_2_ above 0.4%, and a low PaO_2_/FiO_2_ ratio (<195) (Table 1). Additional standard laboratory tests are listed in Supplementary Materials Table S1.

Potential explanations for the high percentage of patients not having fever include Gram-negative infections in 32 patients and fungal infections in 12 patients.

As shown in Table 2, significant variations during the specified time intervals were registered for IL-6, suPAR, PCT, and hsCRP.

During the first two days of observation, 20 patients did not survive. The median IL-6 level in these non-survivors was 707.85 pg/mL (IQR 365.5–864.57), significantly higher than those reported for the patients included in this study. Additionally, the mean suPAR level in these non-survivors was 8348.6 pg/mL (SD ± 2195.35), which was consistent with the values observed in this study.

The ability to predict 28-day mortality of selected biomarkers based on ROC analysis and their cutoff levels are shown in Table 3 and Table 4.

At the initial assessment (T_0_), suPAR demonstrated the highest sensitivity at 85%, followed by IL-6 and hsCRP, both at 80%. At T_24_, the sensitivity of IL-6 increased to 90%, while at T_48_, PCT achieved the highest sensitivity at 95%.

In terms of specificity, hsCRP achieved the highest value at T_24_ with 92.59%. At T_0_, AZU1 had a specificity of 81.48%. At T_24_, AZU1 reached 88.89%, followed by suPAR at 81.48%. By T_48_, suPAR specificity increased to 88.89%, while hsCRP specificity decreased to 81.48%.

Among the biomarkers, suPAR proved to be the most reliable predictor of 28-day mortality, AUC = 0.81 (*p* < 0.001), at T_24_. However, at T_48_, the AUC of suPAR decreased to 0.73 (*p* = 0.002), with similar performances observed for IL-6 (AUC = 0.72, *p* = 0.004) and PCT (AUC = 0.70, *p* = 0.006).

**Figure 2:**
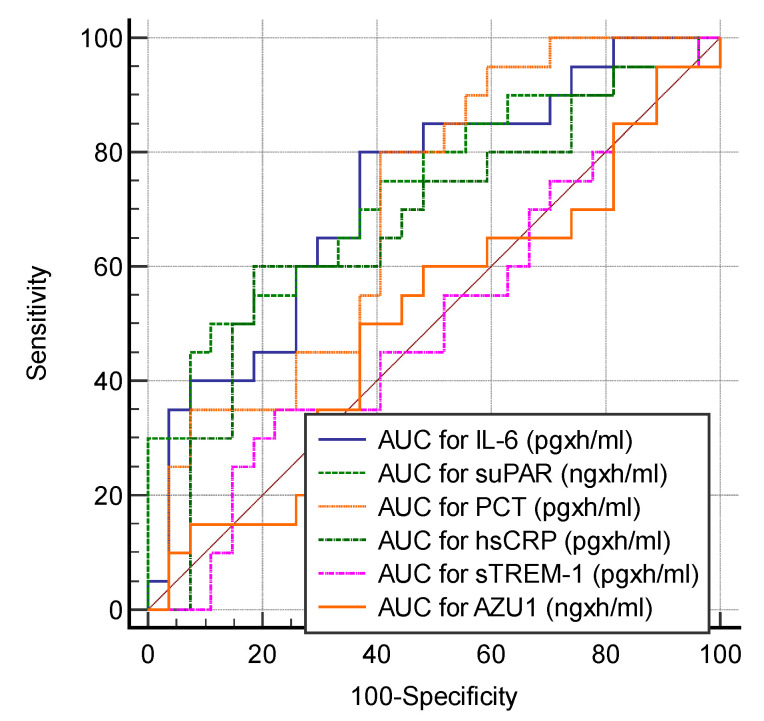
AUCs for study biomarkers.

The AUC obtained with three consecutive measurements at T_0_, T_24_, and T_48_ did not surpass the AUC from a single biomarker evaluation. The highest AUCs were observed with suPAR (0.733, *p* = 0.002), IL-6 (0.730, *p* = 0.002), and PCT (0.700, *p* = 0.009) (Figure 2).

In terms of sensitivity, both PCT and IL-6 achieved 80%. For specificity, suPAR reached 88.89%, followed by hsCRP at 81.48%.

## 4. Discussion

The incidence of sepsis is increasing, particularly in an aging population, with more severe cases seen in the ED due to rising bacterial resistance and the presence of comorbidities [24]. Sepsis is associated with high morbidity and mortality, making timely management and early intervention critical [1]. Given the 42% mortality rate and the median ages of survivors (71.5 years) and non-survivors (74.5 years), age appears to be a significant factor in mortality risk [25,26,27]. The high death rate in this study group may be explained by the combination of advanced age, comorbidities, prolonged inflammation, and the fact that 70% of non-survivors experienced septic shock [25,28].

Biomarkers play an important role in early diagnosis, guiding treatment decisions, and improving prognosis estimation, thereby supporting effective resource allocation [3,29]. This has led to ongoing research to identify new modern biomarkers, reliable ones which in the future can prove their usefulness in real life, at the patient’s bedside, as close as possible to the concept of the “ideal biomarker”.

In this study, we assessed six different biomarkers, suPAR, IL-6, PCT, hsCRP, sTREM, and AZU1, from the point of view of variability over time and their ability to predict mortality at 28 days.

suPAR is recognized as a proinflammatory marker with a significant role in immune system activation. Elevated suPAR levels have been linked to increased disease severity and higher readmission rates in intensive care settings [30]. In our study, suPAR was an independent predictor of 28-day mortality, achieving the highest AUC of 0.813 at T_24_, with a sensitivity of 75%, specificity of 81.48%, and was statistically significant.

Similar findings to our results were reported in a systematic review and meta-analysis conducted by Huang et al. [13] which found that suPAR had a sensitivity of 74% and specificity of 70% for predicting mortality in sepsis. Another study by Nasr El-Din et al. [12] reported an AUC of 0.998 (95% CI, 0.92–1) for suPAR, measured on day seven in septic patients. Given these results, along with suPAR’s remarkable stability over time (estimated to have a half-life of over 19 h and extending up to 7–10 days) [31], suPAR may be a promising biomarker for predicting early mortality in sepsis patients, even beyond the first week of hospitalization [11,12,13].

IL-6 is a key proinflammatory cytokine extensively studied for its role as an independent predictor of sepsis mortality [5,16,32,33]. It plays a critical role in the innate immune response by enhancing monocyte and neutrophil sensitivity and boosting NK cell cytotoxicity [15,32]. In healthy adults, IL-6 concentrations typically range from 0 to 43.5 pg/mL [34], but in sepsis or septic shock, levels can exceed 3500 pg/mL [32]. In our study, IL-6 levels were significantly higher in non-survivors (441.6 pg/mL) and were identified as an independent predictor of 28-day mortality, consistent with previous findings [7,33]. The best mortality prediction values for IL-6 in our study were observed at T_48_, where the AUC reached 0.72. At this time point, sensitivity was 80% and specificity was 62.96%, with a statistically significant *p*-value. It was reported in the literature that IL6R blockade may improve outcome and decrease mortality in sepsis. The use of tocilizumab has proved efficacy in managing cytokine storms during COVID-19 [35,36,37]. There are also studies for other inflammatory conditions, including acute pancreatitis, where targeting the IL-6 pathway may reduce excessive inflammatory response [38].

PCT is one of the most widely used biomarkers in clinical practice, particularly for early diagnostic, prognostic, and guiding antibiotic cessation [1]. Primarily responsible for regulating blood calcium levels, it is produced by thyroid C-cells. In healthy individuals, PCT is present in very low concentrations (<0.05 ng/mL) in the blood, but its levels can increase significantly in response to cytokine stimulation [32]. In our study, the most accurate outcome prediction was observed at T_48_, with an AUC of 0.706. The optimal cutoff value was >2.4, yielding a significant sensitivity of 94% and a specificity of 40.74%, higher than reported in previous studies [8,32].

hs-CRP is an acute-phase protein, and it is well-known as a reliable marker of inflammation. It has been demonstrated to exhibit a substantial increase in concentration within 6–8 h following infection [39], making it a useful tool for the timely diagnosis of sepsis [32]. Although in our study hsCRP demonstrated high specificity, with values of 92.59% at T_24_ and 81.48% at T_48_, its effectiveness in predicting mortality is limited. In comparison, a study by Ling et al. [5] found that although hs-CRP shows high sensitivity for sepsis diagnosis, its utility in predicting mortality is less robust compared to PCT.

To our knowledge, this study is the first to evaluate these novel specific biomarkers within the first two days after a sepsis or septic shock diagnosis in predicting mortality. However, our study has some limitations. This is a monocentric study, which limits the generalizability of the findings. This study was conducted in a single center, and the results may not be representative of broader populations or different healthcare settings. Second, this study was conducted during the COVID-19 pandemic, which imposed several constraints. Our access to patients in hospitals was often restricted, and patient participation was further hindered by their reluctance and fear of exposure to the virus. This resulted in delayed presentations to the ED. These factors reduced the potential sample size, limiting the robustness of our conclusions. However, our sample size is similar to other studies on this topic. It is also possible that if we had included all patients, the predictive value of biomarkers may have been changed. We did not intend in our study to corelate suPAR levels or other biomarkers levels with bacterial strain, despite the fact that there are some publications on this.

## 5. Conclusions

SuPAR may be a promising novel biomarker for predicting short-term mortality in sepsis. IL-6 and PCT are effective predictive biomarkers for 28-day mortality in patients with sepsis and septic shock initially admitted to the ED. To validate these results and better understand the broader applicability of these biomarkers, further large-scale, multicentric studies are needed.

## Figures and Tables

**Table 1:** A comparison between survivors and non-survivors of baseline characteristics, scores, and comorbidities.

Parameters	Time	Survivors (n = 27)	Non-Survivors (n = 20)	*p*
**Age (years) †**	T_0_	71.5 (63.25–81)	74.5 (64.25–82.5)	0.45
**BMI n (%) †**	T_0_	27.56 (23.43–33.53)	25.29 (22.05–30.52)	0.16
***Vital signs and physiological parameters***				
**GCS †**	T_0_	15 (13–15)	11.5 (13–15)	**0.004**
	T_24_	15 (12–15)	10.5 (4.5–2.5)	**<0.001**
	T_48_	14 (12–15)	8 (4–10)	**<0.001**
**Respiratory rate** **(resp/min) †**	T_0_	27 (24–30.75)	30 (26–34.75)	0.20
	T_24_	25 (22.5–30)	25.5 (16–32.75)	0.64
	T_48_	25 (23–28)	22 (18–30)	0.18
**Heart Rate** **(beats/min) †**	T_0_	108 (92.5–112.75)	116 (107.75–126)	0.11
	T_24_	101 (72.5–110)	107 (88.25–121.25)	0.05
	T_48_	102 (70–114)	110 (105–128)	**0.005**
**Glycemia (mg/dl) †**	T_0_	186 (117.5–235.5)	121 (99.25–190.5)	0.02
	T_24_	118.50 (105–183.25)	115.50 (79.25–210.25)	0.30
	T_48_	133 (106–170)	122 (80–215.70)	0.87
**MAP (mmHg) ††**	T_0_	68.2 ± 15.5	60.6 ± 9.9	0.06
	T_24_	76.8 ± 16	73.8 ± 14.9	0.50
	T_48_	75.5 ± 14.8	67.6 ± 9.7	0.04
**Lactate (mmol/L) †**	T_0_	2 (1.52–3.27)	2.85 (1.72–5.6)	0.15
	T_24_	1.7 (1.10–2.22)	2.5 (1.55–3.40)	0.19
	T_48_	1.60 (1–2)	2 (1.8–6)	**0.003**
**SaO_2_ (%) †**	T_0_	94.5 (92–97)	92.5 (86–96)	0.40
	T_24_	96 (94.25–97)	96 (92.75–98.5)	0.81
	T_48_	95 (94–98)	96 (90–99)	0.54
**FiO_2_ (%) †**	T_0_	0.21 (0.21–0.55)	0.6 (0.21–0.87)	0.24
	T_24_	0.21 (21–40)	0.45 (40–71)	**0.001**
	T_48_	0.21 (21–40)	0.40 (35–60)	**<0.001**
**PaO_2_/FiO_2_ †**	T_0_	390.70 (315.87–459.75)	267.55 (109.28–429.82)	0.11
	T_24_	371 (291.25–433.25)	176 (102.14–241.25)	**<0.001**
	T_48_	333 (225–423)	195 (108–288)	**<0.001**
**Temperature (°C) †**	T_0_	37.65 (36.32–38.8)	37.40 (36.6–38.15)	0.22
	T_24_	37 (36.42–37.87)	37 (36.15–37.2)	0.79
	T_48_	36 (36–37.5)	36.5 (36–37)	0.67
***Scores***				
**SOFA †**	T_0_	5 (2.25–9.75)	9.5 (5.50–12.75)	0.06
	T_24_	6 (3–9)	11 (8–14.25)	**0.001**
	T_48_	5 (2–7)	10 (7.25–14)	**<0.001**
**APACHE II ††**	T_0_	18.8 ± 5.6	26 ± 7.7	**0.01**
	T_24_	14.6 ± 5.1	24.9 ± 7.1	**<0.001**
	T_48_	13.1 ± 6.8	24.4 ± 8	**<0.001**
**SAPS II †**	T_0_	46 (39.25–55.50)	59.5 (56–78)	0.001
	T_24_	44 (35.5–49)	63.5 (48–79)	**0.002**
	T_48_	39 (33–51)	62 (41–84)	**0.01**
***Comorbidities, n (%)***				
**Cardiovascular disease**	T_0_	24 (82.8)	15 (88.2)	1.00
**Diabetes**	T_0_	18 (62.1)	9 (52.9)	0.76
**Chronic kidney disease**	T_0_	7 (24.1)	3 (17.6)	0.88
**Chronic lung disease**	T_0_	9 (31)	4 (23.5)	0.83
**Obesity**	T_0_	13 (44.8)	13 (76.5)	0.25
**Neuropsychiatry**	T_0_	11 (37.9)	11 (64.7)	0.14

Legend: † median (IQR), IQR: interquartile range, †† mean ± standard deviation, BMI: body mass index, GCS: Glasgow Coma Scale, MAP: mean arterial pressure, SaO_2_-oxygen saturation, PaO_2_/FiO_2_-ratio of arterial oxygen partial pressure to fractional inspired oxygen, SOFA-Sequential Organ Failure Assessment, APACHE II: Acute Physiology and Chronic Health Evaluation II, SAPS II and III: Simplified Acute Physiology Score II and III.

**Table 2:** Mean and median serum levels of biomarkers measured over the first two days after arrival in the ED.

Biomarker (Plasma Levels)	Time	Survival Group (n = 29)	Non-Survival Group (n = 17)	*p*
**IL-6 (pg/mL) †**	T_0_	406.50 (91.22–535.07)	441.60 (304.90–791.05)	0.11
	T_24_	129.85 (67.70–369.82)	402.10 (245.95–669.60)	**0.003**
	T_48_	75.60 (40.87–213.12)	238.70 (117.95–531.55)	**0.001**
**suPAR (ng/mL) ††**	T_0_	7343.8 ± 1971.1	8512.1 ± 1848.4	0.04
	T_24_	6556.3 ± 1809	8641.8 ± 1765.3	**<0.001**
	T_48_	6405.4 ± 2020.7	8318.9 ± 2449.1	**0.005**
**PCT (pg/mL) †**	T_0_	13.85 (2.87–31.17)	23.10 (7.95–58.15)	0.13
	T_24_	9.95 (3.67–38.82)	21.7 (8.30–81.35)	0.11
	T_48_	6 (1.75–19.72)	15.6 (6.45–71.05)	**0.01**
**hsCRP (pg/mL) †**	T_0_	26.05 (15.20–29.67)	18.40 (16.50–22.40)	0.11
	T_24_	17.90 (14.6 –23.7)	21.5 (15.61–26.72)	0.52
	T_48_	22.80 (20.32–28.85)	17 (14.10–21.75)	**0.01**
**sTREM-1 (pg/mL) †**	T_0_	264.75 (89.80–741.50)	224.50 (119.6–813.90)	0.50
	T_24_	229.75 (110.47–474.72)	341.60 (77.90–555.70)	0.57
	T_48_	175.95 (63.72–467.10)	184.70 (65.90–551.60)	0.69
**AZU1 (ng/mL) †**	T_0_	8.30 (7.55–9.07)	7.30 (7.00–8.60)	0.09
	T_24_	7.80 (6.82–8.57)	7.60 (7.10–9.50)	0.82
	T_48_	7.95 (6.95–9.12)	7.60 (6.90–9.15)	0.63

Legend: † median (IQR), IQR: interquartile range, †† mean ± standard deviation, IL-6: interleukin-6, suPAR: soluble urokinase plasminogen activator, PCT: procalcitonin, hsCRP: high-sensitivity C-reactive protein, sTREM-1: soluble triggering receptor expressed on myeloid cells-1, AZU-1: azurocidin 1.

**Table 3:** AUC and cutoff values of tested biomarkers.

	Time	AUC (95% CI)	Cutoff Values	Se (95% CI)	Sp (95% CI)	*p*
**Biomarkers**						
**IL-6 (pg/mL)**	T_0_	0.630 (0.476–0.766)	>246.6	80 (56.3–94.3)	48.15 (28.7–68.1)	0.11
	T_24_	0.698 (0.547–0.823)	>109	90 (68.3–98.8)	44.44 (25.5–64.7)	**0.009**
	T_48_	0.720 (0.570–0.841)	>96.6	80 (56.3–94.3)	62.96 (42.4–80.6)	**0.004**
**suPAR (ng/mL)**	T_0_	0.695 (0.544–0.821)	>7434	85 (62.1–96.8)	59.26 (38.8–77.6)	0.01
	T_24_	0.813 (0.672–0.912)	>8168	75 (50.9–91.3)	81.48 (61.9–93.7)	**<0.001**
	T_48_	0.731 (0.581–0.849)	>8465	50 (27.2–72.8)	88.89 (70.8–97.6)	**0.002**
**PCT (pg/mL)**	T_0_	0.595 (0.442–0.736)	>19.8	50 (27.2–72.8)	70.37 (49.8–86.2)	0.26
	T_24_	0.662 (0.509–0.793)	>10	75 (50.9–91.3)	59.26 (38.8–77.6)	0.04
	T_48_	0.706 (0.556–0.830)	>2.4	95 (75.1–99.9)	40.74 (22.4–61.2)	**0.006**
**hsCRP (pg/mL)**	T_0_	0.591 (0.438–0.732)	>24.9	80 (56.3–94.3)	48.15 (28.7–68.1)	0.59
	T_24_	0.551 (0.399–0.696)	>18	30 (11.9–54.3)	92.59 (75.7–99.1)	0.55
	T_48_	0.551 (0.517–0.800)	>18.1	60 (36.1–80.9)	81.48 (61.9–93.7)	**0.04**
**sTREM-1 (pg/mL)**	T_0_	0.554 (0.402–0.699)	>189	70 (45.7–88.1)	51.85 (31.9–71.3)	0.53
	T_24_	0.509 (0.359–0.658)	>429.8	40 (19.1–63.9)	74.07 (53.7–88.9)	0.91
	T_48_	0.504 (0.354–0.653)	>70.7	35 (15.4–59.2)	77.78 (57.7–91.4)	0.96
**AZU1 (ng/mL)**	T_0_	0.608 (0.455–0.747)	>7.3	45 (23.1–68.5)	81.48 (61.9–93.7)	0.20
	T_24_	0.507 (0.358–0.656)	>9	35 (15.4–59.2)	88.89 (70.8–97.6)	0.93
	T_48_	0.520 (0.368–0.670)	>7.8	60 (36.1–80.9)	53.85 (33.4–73.4)	0.82

Legend: AUC: area under the curve, CI: confidence interval, Se: sensitivity, Sp: specificity, IL-6: interleukin-6, suPAR: soluble urokinase plasminogen activator, PCT: procalcitonin, hsCRP: high-sensitivity C-reactive protein, sTREM-1: soluble triggering receptor expressed on myeloid cells-1, AZU1: azurocidin 1; receiver operating characteristic (ROC) analysis.

**Table 4:** AUC and cutoff values of biomarkers T_0_–T_48_.

	Time	AUC (95% CI)	Cutoff Values	Se (95% CI)	Sp (95% CI)	*p*
**Biomarkers**						
**AUC for IL-6 (pgxh/mL)**	T_0_–T_48_	0.730 (0.580–0.849)	>180	80 (56.3–94.3)	62.96 (42.4–80.6)	0.002
**AUC for suPAR (ngxh/mL)**	T_0_–T_48_	0.733 (0.584–0.852)	>13,558	50 (27.2–72.8)	88.89 (70.8–97.6)	0.002
**AUC for PCT (pgxh/mL)**	T_0_–T_48_	0.700 (0.549–0.825)	>10.03	80 (56.3–94.3)	59.26 (38.8–77.6)	0.009
**AUC for hsCRP (pgxh/mL)**	T_0_–T_48_	0.670 (0.518–0.800)	>30	60 (36.0–80.9)	81.48 (61.9–93.7)	0.04
**AUC for sTREM-1 (pgxh/mL)**	T_0_–T_48_	0.504 (0.354–0.653)	>119.63	35 (15.4–59.2)	77.78 (57.7–91.4)	0.96
**AUC for AZU1 (ngxh/mL)**	T_0_–T_48_	0.506 (0.356–0.655)	>12.23	50 (27.2–72.8)	62.96 (42.4–80.6)	0.94

Legend: AUC: area under the curve, CI: confidence interval, Se: sensitivity, Sp: specificity, IL-6: interleukin-6, suPAR: soluble urokinase plasminogen activator, PCT: procalcitonin, hsCRP: high-sensitivity C-reactive protein, sTREM-1: soluble triggering receptor expressed on myeloid cells-1, AZU1: azurocidin 1; receiver operating characteristic (ROC) analysis.

## Data Availability

The data presented in this study are available on reasonable request from the corresponding author.

## Supplementary Materials

The following supporting information can be downloaded at: https://www.mdpi.com/article/10.3390/jcm13195678/s1, Table S1: Standard Blood Tests.
