# Supplementary material for: Biomarkers as Predictors of Mortality in Sepsis and Septic Shock for Patients Admitted to Emergency Department: Who Is the Winner? A Prospective Study
Source: J Clin Med. 2024 Sep 24;13(19):5678. doi: 10.3390/jcm13195678 (PMC11477125; doi:10.3390/jcm13195678)
Supplement: Supplementary file 1 [file jcm-13-05678-s001.zip › jcm-3205857-supplementary.pdf]

Table S1. Standard Blood Tests

Parameters [median (IQR)]	Time	Survival group (n = 27)	Non-survival group (n = 20)	*P*
**Complete Blood Count**				
**WBC (×10⁹/L)**	T_0	15.76 (11.28 - 23.51)	16.49 (6.41 - 20.06)	0.473
	T_{24}	13.13 (7.98 - 17.76)	19.61 (9.01 - 30.98)	0.393
	T_{48}	12.48 (9.37 - 17.72)	14.39 (9.74 - 22)	0.300
**Platelets (×10⁹/L)**	T_0	182 (125 - 234.25)	202 (22.25 - 269.5)	0.674
	T_{24}	144.50 (116.75 - 189)	180.50 (134 - 264)	0.432
	T_{48}	155 (106 - 234)	162 (123 - 190)	0.891
**Hemoglobin (g/dL)**	T_0	11.75 (10.72 - 13.5)	10.25 (9.62 - 12.2)	0.056
	T_{24}	11.40 (9.22 - 12.57)	9.40 (8 - 12.25)	0.106
	T_{48}	10.90 (9.90 - 11.80)	9.30 (7.90 - 12)	0.064
**Hematocrit (%)**	T_0	34.85 (30.92 - 39.3)	31.40 (28.22 - 35.77)	0.127
	T_{24}	34.50 (27.95 - 37.05)	29.85 (23.77 - 36.07)	0.094
	T_{48}	32.90 (29.30 - 34.70)	28.80 (22.60 - 35)	0.042
**Neutrophil (×10⁹/L)**	T_0	13.52 (10 - 21.84)	13.86 (5.85 - 18.51)	0.488
	T_{24}	11.55 (7.13 - 15.44)	17.65 (7.77 - 28.26)	0.387
	T_{48}	10.81(6.97 - 15.15)	11.49 (8.32 - 20)	0.191
**Lymphocyte (×10⁹/L)**	T_0	0.93 (0.62 - 1.61)	0.55 (0.22 - 1.01)	0.018
	T_{24}	1.04 (0.71 - 1.53)	0.81 (0.52 - 1.36)	0.082
	T_{48}	0.98 (0.74 - 1.52)	0.73 (0.44 - 2)	0.162
**Monocyte (×10⁹/L)**	T_0	0.54 (0.35 - 1.08)	0.61 (0.31 - 0.93)	0.641
	T_{24}	0.60 (0.32 - 0.86)	0.53 (0.21 - 0.99)	0.633
	T_{48}	0.61 (0.41 - 0.91)	0.65 (0.40 - 0.78)	0.750
**MPV (fL)**	T_0	10.6 (9.75 - 12.57)	10.15 (8.2 - 11.82)	0.311
	T_{24}	10.60 (9.35 - 11.62)	10.60 (9.35 - 11.62)	0.198
	T_{48}	11.20 (9.80 - 12.60)	10.40 (8.90 - 11)	0.073
**LMR**	T_0	1.28 (0.92 - 3.29)	1.08 (0.71 - 1.59)	0.215
	T_{24}	1.77 (1.18 - 3.74)	1.52 (0.93 - 3.50)	0.502
	T_{48}	2 (1.22 - 4.46)	1.32 (0.83 - 2.4)	0.097
**NLR**	T_0	14.58 (8.50 - 24.22)	20.44 (15.11 - 26.12)	0.176

	T_{24}	9.87 (5.33 - 18.27)	18.78 (10.19 - 25.68)	0.199
	T_{48}	9.29 (6.62 - 18.28)	14.38 (9.90 - 26.97)	0.082
**PLR**	T_0	173.33 (105.33 – 270.56)	261.31 (156.43 - 559.13)	0.013
	T_{24}	140.56 (71.37 - 225.21)	182.75 (104.63 - 301.47)	0.215
	T_{48}	239.51	208.69	0.260
**SII**	T_0	2387.31 (916.64 - 4609.85)	3765.84 (1903.84 - 6088.18)	0.207
	T_{24}	1730.15 (604.99 - 3745.50)	3231.40 (1307.60 - 5322.72)	0.207
	T_{48}	1450.98 (702.14 - 2591.58)	2238.09 (1740 - 4045.94)	0.199
**SIRI**	T_0	10.13 (3.45 - 20.91)	11.51 (7.61 - 15.57)	0.759
	T_{24}	6.69 (0.05 - 11.65)	7.30 (4.80 - 21.81)	0.517
	T_{48}	5.57 (2.65 - 10.59)	9 (5.93 - 17.78)	0.082
**Coagulation**				
**INR**	T_0	1.39 (1.27 - 1.62)	1.42 (1.13 - 1.77)	0.669
	T_{24}	1.32 (1.21 - 1.52)	1.65 (1.35 - 1.80)	0.168
	T_{48}	1.36 (1.23 - 1.52)	1.51 (1.43 - 2.20)	0.116
**PT (sec)**	T_0	15.75 (17.07 - 21.8)	16.10 (12.77 - 20.82)	0.696
	T_{24}	14.55 (13.92 - 15.65)	17.45 (14.65 - 19.90)	0.148
	T_{48}	15.20 (14.10 - 17.90)	17.50 (15 - 25)	0.032
**PI (%)**	T_0	59.50 (38.75 - 65.8)	61.20 (42.75 - 84)	0.469
	T_{24}	63.50 (51.75 - 72.75)	56.50 (51.72 - 62.80)	0.593
	T_{48}	60 (54 - 71)	55 (45 - 69.20)	0.381
**APTT (sec)**	T_0	28.40 (25.62 - 33.67)	27.60 (25.95 - 36.7)	0.794
	T_{24}	30.20 (27.15 - 33.65)	30.50 (27.35 - 34.57)	0.638
	T_{48}	32.10 (26 - 36.20)	37 (28.20 - 42)	0.085
**Liver function**				
**ALT (U/L)**	T_0	25 (14.5 - 43)	25 (13 - 77.25)	0.864
	T_{24}	25.50 (15.25 - 38)	22 (12.75 - 55.75)	0.864
	T_{48}	27 (19 - 33)	23 (18 - 267)	0.412
**AST (U/L)**	T_0	36.50 (19.25 - 62)	40 (21.25 - 124.75)	0.495
	T_{24}	38.50 (19 - 64.75)	34 (22 - 81.25)	0.649
	T_{48}	31 (19 - 51)	42 (27.50 - 572)	0.035
**BT (mg/dl)**	T_0	0.84 (0.58 - 1.26)	0.88 (0.59 - 1.55)	0.554
	T_{24}	0.67 (0.44 - 0.80)	0.76 (0.49 - 1.31)	0.145

	T_{48}	0.54 (0.40 - 1.02)	0.92 (0.68 - 1.80)	0.011
**BD (mg/dl)**	T_0	0.26 (0.15 - 0.51)	0.44 (0.19 - 0.85)	0.210
	T_{24}	0.23 (0.13 - 0.38)	0.50 (0.30 - 0.94)	0.012
	T_{48}	0.30 (0.13 - 0.51)	0.42 (0.31 - 0.87)	0.083
**Kidney function**				
**Urea (mg/dl)**	T_0	101 (66.5 - 215.25)	118 (78 - 199.5)	0.982
	T_{24}	94 (54.75 - 191.96)	152 (86.87 - 221)	0.569
	T_{48}	90 (55 - 146)	150 (102.30 - 205)	0.106
**Creatinine (mg/dl)**	T_0	2.69 (1.65 - 4.65)	2.69 (1.46 - 3.98)	0.295
	T_{24}	1.87 (1.32 - 3.39)	2.49 (1.44 - 4.07)	0.593
	T_{48}	1.38 (1 - 2.4)	2.27 (1.18 - 3.74)	0.074
**eGFR (ml/min/1.73 m^2)**	T_0	22.20 (10.89 - 39.47)	23.29 (14.07 - 35.02)	0.577
	T_{24}	31.55 (18.94 - 51.17)	25.35 (14.42 - 30.60)	0.432
	T_{48}	42.40 (22 - 60.83)	21 (14 - 47.80)	0.043
**ABG**				
**pH**	T_0	7.42 (7.28 - 7.48)	7.35 (7.22 - 7.47)	0.406
	T_{24}	7.36 (7.32 - 7.43)	7.32 (7.25 - 7.44)	0.509
	T_{48}	7.39 (7.34 - 7.43)	7.32 (7.26 - 7.48)	0.083
**PO_2 (mmHg)**	T_0	85 (76.42 - 102.15)	81.60 (70.32 - 106.72)	0.609
	T_{24}	87.50 (73.10 - 109.25)	87.05 (59.52 - 96)	0.357
	T_{48}	89 (73 - 98)	90 (72 - 106)	0.690
**PCO_2 (mmHg)**	T_0	27.70 (22.65 - 31.57)	24.40 (20.02 - 24.40)	0.426
	T_{24}	28.05 (25.50 - 32)	28.60 (25.90 - 30.10)	0.855
	T_{48}	26 (24 - 32)	28 (25.6 - 31.4)	0.284
**FiO_2 (%)**	T_0	0.21 (0.21 - 0.55)	0.6 (0.21 - 0.87)	0.242
	T_{24}	0.21 (21 - 40)	0.45 (40 - 71)	**0.001**
	T_{48}	0.21 (21 - 40)	0.40 (35 - 60)	**<0.0001**
**Na^+ (mmol/L)**	T_0	134 (127.92 - 136.90)	137.05 (125.95 - 140.6)	0.554
	T_{24}	139 (135 - 142.75)	139.75 (131.6 - 147.25)	0.624
	T_{48}	137.20 (132 - 142)	140 (134.7 - 150)	0.419
**K^+ (mmol/L)**	T_0	4.33 (3.94 - 5.04)	4.36 (3.17 - 6.10)	0.842
	T_{24}	3.92 (3.61 - 4.65)	4.57 (3.60 - 5.27)	0.909
	T_{48}	4.20 (3.80 - 4.50)	4.26 (3.80 - 4.80)	0.829

**HCO_3^-(mmol/L)**	T_0	18.40 (12.64 - 21.82)	13.70 (10.92 - 18.22)	0.145
	T_{24}	19 (15.36 - 20.17)	15.95 (13.75 - 19.16)	0.260
	T_{48}	20 (13.20 - 22)	18 (14.42 - 20)	0.087
**Cl^- (mmol/L)**	T_0	105.20 (101.7 - 111.07)	110 (100.4 - 114.97)	0.155
	T_{24}	108.50 (104 - 112.15)	114.10 (106.30 - 118.50)	0.363
	T_{48}	108 (102 - 110.30)	110 (106 - 113)	0.189
**Lactate (mmol/L)**	T_0	2 (1.52 - 3.27)	2.85 (1.72 - 5.6)	0.158
	T_{24}	1.7 (1.10 - 2.22)	2.5 (1.55 - 3.40)	0.198
	T_{48}	1.60 (1 - 2)	2 (1.8 - 6)	**0.003**
**BE (mmol/L)**	T_0	-7.1 (-13.7 - -1.82)	-13.35 (-17.27 - -6.42)	0.172
	T_{24}	-6.45 (-10.07 - -1.22)	-8.60 (-13.10 - -5.10)	0.459
	T_{48}	-5.10 (-10 - -1.30)	-9.30 (-10.50 - -3)	0.158
**AG (mmol/L)**	T_0	14 (9.7 - 16.75)	15.50 (6.87 - 18.82)	0.953
	T_{24}	12.95 (9.92 - 16.75)	12.50 (7.15 - 15.52)	0.419
	T_{48}	13 (8.80 - 16.70)	12.90 (5.60 - 17)	0.750
**PaO_2/FiO_2**	T_0	390.70 (315.87 - 459.75)	267.55 (109.28 - 429.82)	0.114
	T_{24}	371 (291.25 - 433.25)	176 (102.14 - 241.25)	**<0.0001**
	T_{48}	333 (225 - 423)	195 (108 - 288)	**<0.0001**

Legend: WBC – White Blood Cells, MPV –Mean Platelet Volume, VEM – Volume of Erythrocytes, LMR – Lymphocyte-to-Monocyte Ratio, NLR – Neutrophil-to-Lymphocyte Ratio, PLR – Platelet-to-Lymphocyte Ratio, SII – Systemic Immune-Inflammation Index, SIRI - Systemic Inflammatory Response Index , INR – International Normalized Ratio, PT – Prothrombin Time, PI - Prothrombin Index, APTT – Activated Partial Thromboplastin Time, ALT – Alanine Transaminase, AST – Aspartate Transaminase, BT – Total Bilirubin , BD – Direct Bilirubin , GFR – Glomerular Filtration Rate, ABG – Arterial Blood Gases, PO2 - Partial Pressure of Oxygen, PCO2 – Partial Pressure of Carbon Dioxide, Na - Sodium, K - Potassium, Cl - Chloride, BE – Base Excess, AG – Anion Gap, PaO2/FiO2 – Ratio of Arterial Oxygen Partial Pressure to Fractional Inspired Oxygen. Data are expressed as median and quartile (25th - 75th percentiles) and analyzed using the Mann-Whitney U Test.
